# Screening for new macrophage therapeutics

**DOI:** 10.7150/thno.34421

**Published:** 2019-10-15

**Authors:** Christopher B. Rodell, Peter D. Koch, Ralph Weissleder

**Affiliations:** 1 Center for Systems Biology, Massachusetts General Hospital, 185 Cambridge St, CPZN 5206, Boston, MA 02114,; 2 Department of Systems Biology, Harvard Medical School, 200 Longwood Ave, Boston, MA 02115

**Keywords:** macrophage therapeutics, screening

## Abstract

Myeloid derived macrophages play a key role in many human diseases, and their therapeutic modulation via pharmacological means is receiving considerable attention. Of particular interest is the fact that these cells are i) dynamic phenotypes well suited to therapeutic manipulation and ii) phagocytic, allowing them to be efficiently targeted with nanoformulations. However, it is important to consider that macrophages represent heterogeneous populations of subtypes with often competing biological behaviors and functions. In order to develop next generation therapeutics, it is therefore essential to screen for biological effects through a combination of *in vitro* and *in vivo* assays. Here, we review the state-of-the-art techniques, including both cell based screens and *in vivo* imaging tools that have been developed for assessment of macrophage phenotype. We conclude with a forward-looking perspective on the growing need for noninvasive macrophage assessment and laboratory assays to be put into clinical practice and the potential broader impact of myeloid-targeted therapeutics.

## Macrophage Types and Therapeutics

Phagocytic cells in the tumor microenvironment (TME) are typically myeloid derived cells recruited from the bone marrow 1. Tumor-associated macrophages (TAMs) in particular can diversify into a spectrum of states, with either promoting or limiting tumor functions. At the time of writing, the field remains incompletely understood; however, a “parts list” of molecular cell types is emerging 2. Traditionally, macrophages have been classified as either classically (M1) or alternatively (M2) activated in response to defined stimuli. These phenotypes are associated with anti- and pro-tumor activities, respectively 3. *In vivo*, however, macrophages typically display complex phenotypes that extend well beyond canonical M1 and M2 conventions 2, 4-6. Using single-cell RNA sequencing, new macrophage states have been uncovered 2, 7. Most recently, the Pittet and Klein groups have identified 14 distinct TAM subtypes. In human cancers, the majority of these states are representative of the immunosuppressive M2 phenotype, with few anti-tumoral M1 phenotypes represented 2. In addition, there was limited overlap in myeloid cell population structures between the blood and tumor; hence, profiling of circulating myeloid cells is poorly representative of the TME, motivating the local assessment of TAM phenotypes.

Numerous strategies for manipulation of macrophages have been proposed in the context of cancer immunotherapy. These methods include targeting of selective cell functions (e.g., enzymatic activity, recruitment), TAM depletion, and phenotypic re-polarization. All of these strategies are promising methods for combination with checkpoint immunotherapies 8, and activation of TAMs toward a functional phenotype better suited to preventing tumor growth has decently demonstrated excellent success 9, 10. To date, there are relatively few clinically approved therapeutics that promote anti-tumorigenic polarizations. Various therapeutic strategies have been proposed, including small molecule compounds, nanoformulations, biologics, and vaccines. From a biological perspective, a promising approach is to stimulate pattern recognition receptors (PRRs) that control innate immune pathways, such as toll-like receptor (TLR) and cytosolic nucleic acid sensing pathways 11-14. Activation of these pathways triggers secretion of pro-inflammatory and anti-viral cytokines, indicative of anti-tumorigenic polarizations. Most activators of these pathways are large, complex compounds, such as CpG DNA, lipopeptides, cyclic dinucleotides, double-stranded RNA as well as their synthetic mimetic (poly(I:C)) that require nanoformulations and/or specialized delivery systems. A key exception are imidazoquinolines, which are small molecule TLR agonists. This group has shown that resiquimod, a TLR7/8 agonist, (R848) promotes M1 polarization, and its anti-cancer effects can be enhanced via cyclodextrin nanoparticle delivery 15. Other small molecule agonists of TLRs and STING are beginning to emerge, and other strategies for macrophage activation have been explored, such as inhibition of CSF1R 16, 17. Given the panoply of pharmacological agents, dosages and formulations, screening for biological effects is critical to narrowing down viable therapeutic approaches. In this review, we describe *in vitro*, *in vivo,* and translational approaches useful in the systematic investigation of new myeloid-targeted therapeutics with an emphasis on anti-tumorigenic macrophage activation.

## Cell Based Screens

Robust screening assays will expedite the future discovery of candidate therapeutics. In search of ideal myeloid screens, several variables need to be considered. These include cell type (primary isolate vs. cell line), cell source (human vs. mouse) and assay type (gene expression assays, high-content screens, co-culture screens; **Tables 1-2**). A number of different assays have been described for screening macrophage polarization, with most using genetically engineered reporters 18-20, phenotypic screens 15, 21 or molecular secretion assays 22, 23. Each method has certain advantages and disadvantages that need to be considered when embarking upon a screen. In the subsequent sections, we discuss considerations of cell sourcing and screening methodologies.

### Model Systems for Screening

#### Human Immune Cells

Perhaps the most practical and physiologically relevant models for human disease are primary macrophages derived from peripheral blood mononuclear cells. Peripheral blood mononuclear cells (PBMCs) are typically isolated from a leukopak (using a Ficoll column), and CD14+ monocytes are then purified using either flow or magnetic sorting 24, 25. Once CD14+ monocytes are isolated, macrophages can be obtained by treating the cells with recombinant human M-CSF for one week. These cells are then polarized into either an M1, M2, or other state using the appropriate stimuli 26. The final cells should be adherent, elongated, and are compatible with a number of different assays that are further described below. While these macrophages are not immortalized and thus cannot proliferate, isolation from a single donor can yield millions of cells, allowing for evaluation of hundreds to even thousands of compounds in high-throughput plate formats 23.

A significant liability with primary cells is donor-donor variability, so any drug candidate discovered in a primary cell based screen needs to be verified across multiple donors. Along these lines, primary cells are not necessarily robust. Cells from certain donors may not be very responsive even to strong positive controls, making evaluation of compounds difficult. Lastly, an additional challenge with primary human cells is the lack of translation into a cell line. Any initial finding in a primary human cell type may not necessarily reproduce in a similar cell line or mouse model, complicating further studies of the drug's mechanism and efficacy.

Stem cell derived macrophages are an emergent tool for the study of human macrophage behavior. These cells may be produced by deriving monocytes from human embryonic stem cells (hESC) or human induced pluripotent stem cells (hiPSC) with subsequent M-CSF treatment to differentiate macrophages 27. These models allow for the development of proliferative cell lines and high cell numbers unavailable in patient-derived primary cells. Resultant cells are susceptible to standard polarization procedures. hiPSC-derived monocyte cell lines are compatible with a number of cell based assays, including the development of co-culture systems, discussed below 28.

A key advantage with stem cell derived macrophages is the ability to create genetically modified macrophages lines. Macrophages are very difficult to manipulate genetically. Stem cells, on the other hand, are relatively susceptible to genetic modifications via viral transfection. As such, one may genetically manipulate progenitor stem cells and subsequently create a macrophage cell line with engineered traits. The ability to develop genetically engineered macrophages perpetuates the study of macrophage migration in the TME, such as by fluorescent labeling 29, or examination of therapeutic treatments including induced cytokine expression 30. Despite these advantages, deriving primary macrophages from stem cells are still less common than obtaining cells from blood donors. This is partly due to the fact that this method is more complex and costly, requiring specialized media as well as other reagents. Advances in the stem cell biology field will likely increase the prevalence of this model system in the future.

In addition to primary cells, a number of immortalized human cell lines exist which are readily accessible. One common option is the THP-1 cell line. These cells are CD14+ “monocyte-like” cells derived from an AML patient. THP-1 cells are maintained in suspension culture, but can be differentiated into “macrophage-like” adherent cells through treatment with phorbol 12-myristate 13-acetate (PMA) or M-CSF 16, 31, 32. THP-1 cells can also be skewed towards different polarizations, using protocols similar to those used for primary cells 31. While THP-1 cells lack physiological relevance compared to primary cells, they are easier to handle, can be grown to large volumes, and are more robust. THP-1 cells express several innate immune pathways responsible for promoting anti-tumorigenic polarizations, such as TLR2/4/8, STING, and RIG-I. Thus, screening in a THP-1 cell line allows simultaneous probing of multiple pathways relevant to macrophage polarization. Currently, THP-1 cells are especially convenient screening platforms because several reporter cell lines that secrete luciferase and/or alkaline phosphatase enzymes are now commercially available (InvivoGen). These cell lines are compatible with high-throughput assays, reducing the need for time consuming and costly ELISAs and/or qPCRs in a primary screen. Additionally, several genetic KO cell lines are now commercially available, allowing further dissection of relevant molecular pathways.

There are a number of less common human cell lines that may be useful for screening. These include, for example, U937 cells which are another immune cell line isolated from a histiocytic lymphoma. While similar to THP-1 cells, they are less common and used to study the behavior and differentiation of monocytes. U937 cells differentiate in response to soluble stimuli, adopting the morphology and characteristics of mature macrophages 33. Non-immune cell lines may also have utility in screening. While they cannot be made into macrophages to study polarization directly, some express the aforementioned innate immune pathways relevant in macrophage polarization. BJ-1 fibroblasts are one such example 34.

#### Murine Immune Cells

While human cell sources have a higher degree of clinical relevance, murine cells have the advantage of providing a closed-loop system for experimentation where screening hits may be further evaluated using *in vivo* mouse models of disease. There are several potential sources of murine macrophages, including splenic, peritoneal, and bone-marrow derived macrophages (BMDMs). BMDMs are among the most common. In this method, cells from the bone marrow of femurs and tibias are isolated, and differentiated using M-CSF 35-37. As with human cells, polarization can be tuned with the addition of specific growth factors. Use of primary murine cells confers some unique advantages over human cells. A primary advantage is the ability to isolate cells from genetically engineered mice, including from cytokine reporter mice (e.g., IL-12 or IFNγ reporters, discussed later) such that the genetically engineered marker (e.g. fluorescent protein) can be used directly for an assay readout, forgoing antibody based assays. Furthermore, primary murine cells exhibit little donor variability as compared to primary human cells, and a number of knock-out models exist from which derived cells are a valuable tool for pathway validation.

In the context of cancer immunotherapy, a more physiological relevant model is tumor-associated macrophages (TAMs). Implantation of various tumor lines, such as MC38, into immunocompetent mice causes robust macrophage infiltration. These TAMs can be accessed by flow sorting macrophages (e.g. CD68+ or F4/80+) from resected tumors. TAMs can be seeded directly onto high-throughput plates and treated as in a regular screen. In contrast to BMDMs though, yields for TAMs are much lower.

For long term culture, it is possible to immortalize BMDMs by infecting them with a retrovirus 38. However, there are also immortalized macrophage cell lines, such as RAW264.7, which was derived from a tumor-bearing BALB/c mouse. These cell lines remain a very commonly used model to study macrophage polarization 39. They are an adherent cell line, which can also be polarized towards M1 or M2 phenotypes with various growth factors. Like THP-1 cells, they express several innate immune pathways relevant in macrophage polarizations, allowing for examination of multiple pathways. Convenient reporter lines as well as genetic KOs are now commercially available from InvivoGen. J774.A.1 cells are another macrophage line derived from a BALB/c mouse. Like RAW264.7 cells, J774 cells also express several inflammatory pathways and are responsive to various PRR agonists. An engineered line with enzymatic reporters is available from InvivoGen, though genetic KOs are not readily available.

#### Non-immune Cell Types for Pathway Specific Analysis

Common non-immune cell lines, such as HEK293 and HeLa, have also been used in screening. These cell lines have either low or no expression of immune pathways, thereby requiring receptors, enzymes, and reporters to be overexpressed. Commercial vendors offer various sets of HEK293 reporter cell lines, expressing different pattern recognition receptors, such as STING, TLR2, and TLR8 (InvivoGen). While the host cell line is human, receptors for either human or mouse can be incorporated. Due to the tendency for some of these cells lines to lose expression with passage, care should be taken to follow manufacturer's protocols for positive selection, assay at early passage, and use of positive controls. When performed correctly, these assays efficiently screen for compounds that directly activate a specific receptor of interest.

### Screening Assays

**Figure 1:**
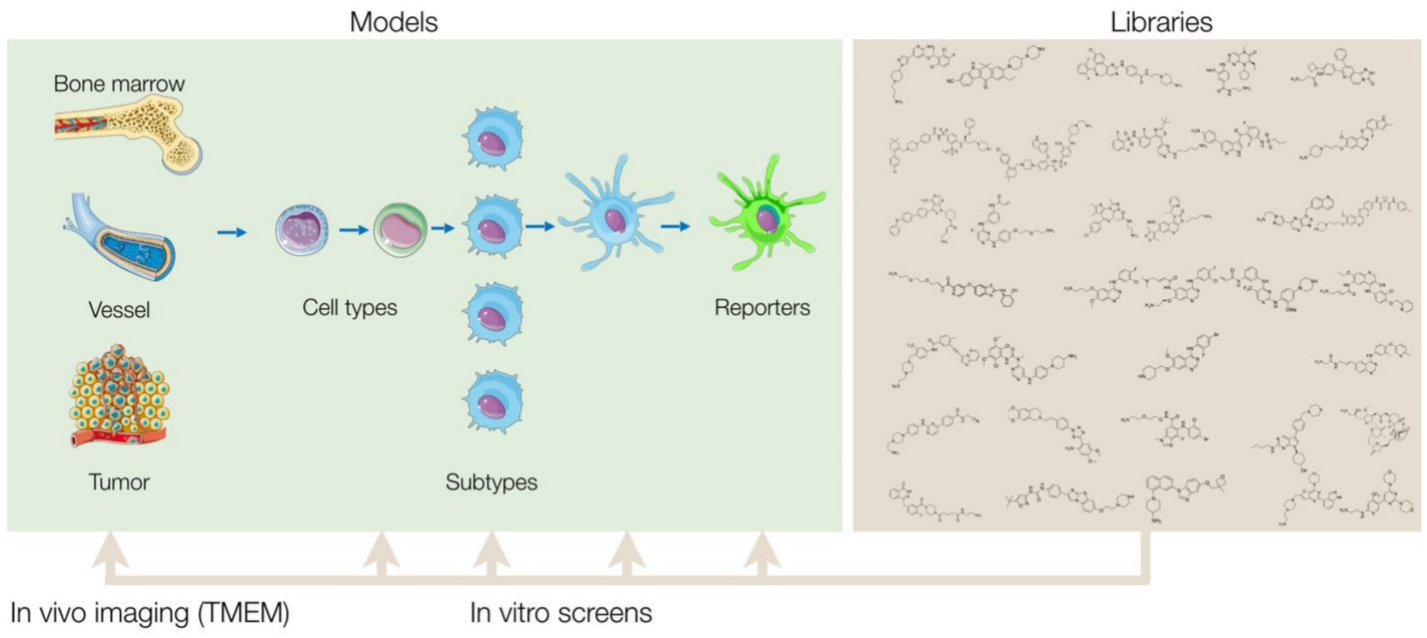
** Overview of screening approach.** Small molecule libraries can be screened in a variety of primary *in vitro* assays that vary based on cell source, subtype, and assay readout. After validation of the results, hits from the primary screen are then tested in additional secondary assays to further characterize the compounds' biological effects. By comprehensively profiling the biological effects of the initial hits, one can then prioritize and narrow down the final list of compounds to be tested *in vivo*. Ultimately, a single or select set of compounds can be administered into mice and other *in vivo* model systems, and a combination of intravital microscopy and various other methods can be used to quantify the therapeutic effect of the compounds in myeloid cells of the tumor microenvironment.

The general pipeline of a screening project is described in **Figure 1.** In any *in vitro* screen, one tests anywhere from tens to thousands of compounds and evaluates their ability to promote a macrophage phenotype of interest. The initial screen is referred to as a primary screen. After conducting a primary screen, hits are then further characterized by studying their effects in additional, secondary screening assays. These assays are distinct from the primary assays and allow one to profile the compounds' mechanisms in more detail. Doing so can aid in prioritizing which compounds have the most therapeutic promise and may shed light on the mechanisms of drug action. Many assays can be used for both primary and secondary screening assays. Some assays may be more appropriate for early phases of a project, while others may be better suited for the later phases. We describe these assays below, and discuss considerations to take into account when designing a screen.

#### Bulk Gene Expression Assays - Genetic Reporters

In bulk gene expression assays, one treats cells with a set of compounds and subsequently evaluates mRNA or protein expression changes, typically on the time scale of 24-48 hours. There are several ways bulk gene expression assays can be implemented in the context of macrophage polarization. Activation of innate immune signaling pathways typically triggers M1 polarization, so most screens assay for pathway activation as a surrogate for general polarization response. Screening for activators of these pathways can most easily be done by using commercial reporter cell lines, which use either luciferase or fluorescent reporters as a readout. These assays are typically very robust. They have high Z-factor (Z') and strictly standardized mean difference (SSMD) scores, which are metrics used in high-throughput screening to measure how well separated positive and negative control groups are. Greater Z-factor or SSMD scores indicate that an assay is more reliable, as hits can be more easily discriminated in a screen. For gene expression assays related to macrophage polarization, an easy option is to take advantage of the commercially available THP-1 and RAW264.7 lines with luciferase reporters. Other published screens have used cell lines with engineered reporters in conceptually identical assays. The discovery of G10, a human-specific STING pathway activator, was made in a screen using fibroblasts with luciferase under control of an interferon stimulated response element (ISRE) 20. Additionally, the HEK-TLR reporter lines were originally developed as a tool for discrimination of imidazoquinoline agonism of human and murine TLR7/8 40, and have more recently been used to validate CU-T12-9 as an agonist of the TLR1/2 heterodimer 41.

#### Bulk Gene Expression Assays - ELISA and qPCR

When primary cells without genetic reporters are investigated, ELISA or qPCR become frequently used read-out methods. Such assays are conceptually identical to those mentioned above, but with the exception that measurements must be made on endogenous mRNA and/or protein. Sandwich ELISAs are excellent for measurements of various cytokines. Compared to qPCR, ELISAs are generally easier, faster, less noisy, and provide a direct read of the amount of protein produced. However, ELISAs are antibody dependent and are not ideal for measurements of intracellular proteins. Moreover, certain cytokines can bind tightly to their extracellular receptors or are difficult to detect in culture media, complicating analysis. qPCRs on the other hand are very sensitive, requiring fewer cells are per assay condition. Species dependence of suitable polarization markers has been established 26.

Similar to ELISAs, Luminex assays are an additional option 22. Conceptually similar to sandwich ELISAs, these assays allow for simultaneous measurement of up to 50 cytokines in a single sample through conjugation of the capture antibodies to color-coded polystyrene beads, while the detection antibody is conjugated to phycoerythrin (via biotin-streptavidin). Using a specialized laser-scanning/flow device, one can measure all levels of all 50 targets using the color-code of the beads along with the PE intensity. This assay type is also high-throughput compatible. Disadvantages are cost and the need for specialized detection equipment. While it also provides a wealth of multi-dimensional data, it is arguably not necessary in a primary screen and more suited to later stages of investigation.

#### Analysis of Macrophage Phenotype by Single Cell RNA-seq (scRNA-seq)

Single cell RNA-seq (scRNA-seq) has emerged as an incredibly powerful tool for analysis of cell types. Its use is particularly prevalent in immunology, where it has been instrumental in refining immune cell types and classifications beyond what has currently been possible via immunohistochemistry and flow cytometry. In cancer immunotherapy, scRNA-seq has been applied heavily to the study of anti-tumor T cells 42, 43, but a thorough characterization of myeloid cells, both in the blood and in the tumor microenvironment, is also emerging 2, 14, 44. As referenced above, our group has shown, with scRNA-seq, that M2 macrophages express varying levels of Arg1, challenging the conventional view that all M2 macrophages are Arg1+ 7. Moreover, we also showed that anti-PD-1 therapy causes depletion of Arg1+ TAMs, thereby suggesting that Arg1 inhibitors would have little utility in combination with anti-PD-1 therapy.

The Arg1 example above highlights just one example of how significant time and effort could be placed on studying a suboptimal biomarker. Hence, characterization of myeloid cell by scRNA-seq could be an important step prior to initiating a screen, in order to establish a relevant biomarker. As an assay to be used directly in an *in vitro* screen, however, scRNA-seq has limited utility due to its low throughput and very high cost. To profile drug activity, its use is more warranted at the later stages of a screening project, in which the effect of one drug needs to be characterized comprehensively across a population of cell types.

#### High-Content Screening (HCS)

High-content screening refers to assays in which several phenotypes can be captured at single cell resolution. It most often refers to screens using high-throughput microscopy as a readout; though, other assays such as flow cytometry and CyTOF can also be employed in high-throughput format 45, 46. In high-content screening, cells of interest are again treated with the compound library and subsequently imaged using one of the above methods. While ELISA/qPCR based assays are often easier and have higher Z' scores than HCS, high-content screening is a more effective approach that can better capture the intricacies of macrophage phenotypes in various model systems.

HCS is most commonly done in standard 96, 384, or 1536 well formats which lend themselves to high-throughput robotic automation. Adherent cells are ideal, though it is also possible to screen in suspension cells using biocompatible adhesive reagents, such as CellTak 47. The most common approach is to fix and immunostain the cells for various markers with fluorescently labeled antibodies and/or dyes. Typically, up to four markers can be simultaneously imaged without concern of spectral overlap. However, various methods are being pursued with the goal of developing higher multiplexed assays in which several more phenotypes can be measured. In cyclic imaging for example 48, 49, one stains cells with a cocktail of markers, signal is bleached or washed away, and cells re-stained with a separate cocktail of markers. These methods allows for detection of as many as 30 markers at once. Other approaches involve barcoding antibodies with DNA, using the sequence information to identify levels of each marker 50, 51.

**Figure 2:**
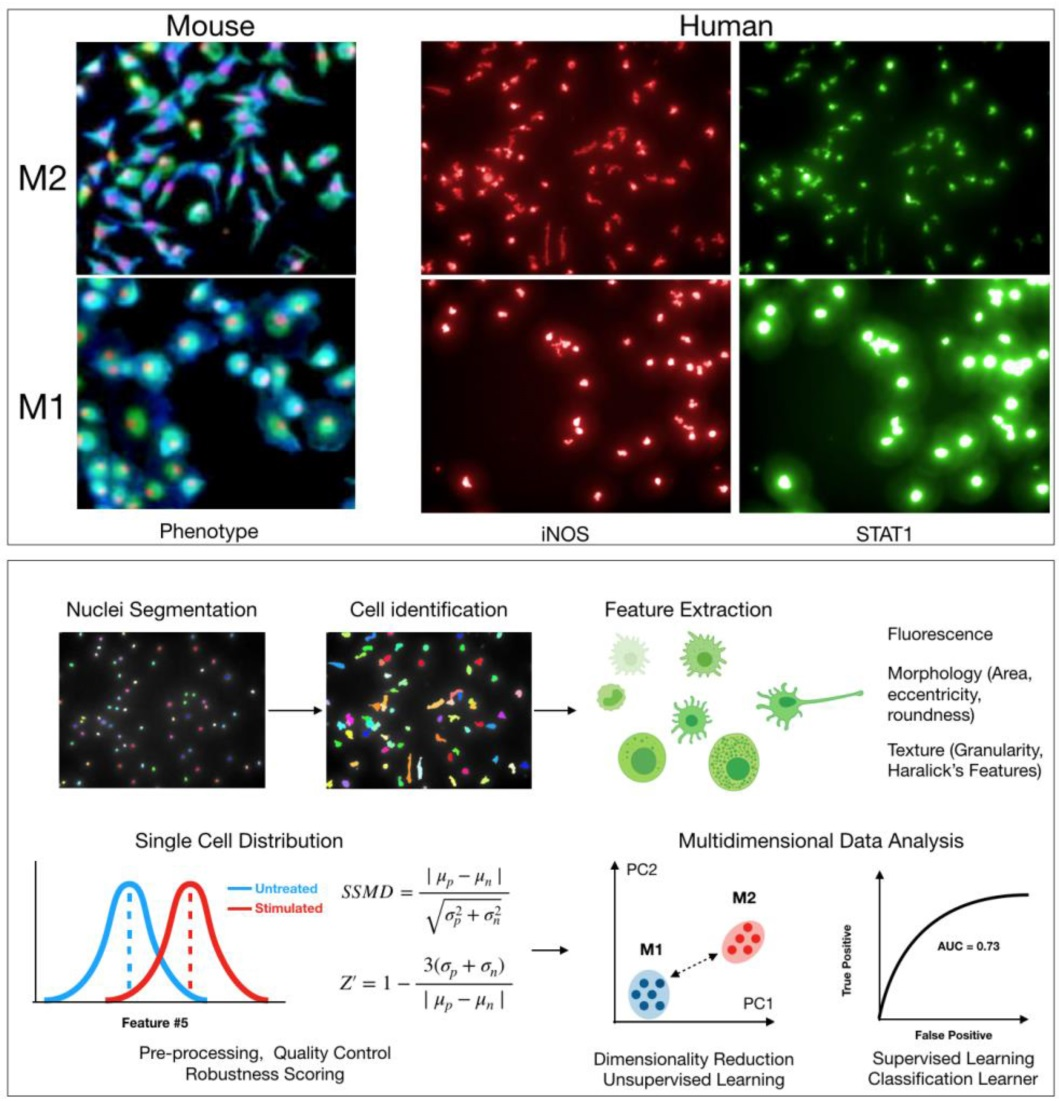
** Cell based screen.** High-content screening allows for quantification of hundreds of features pertaining to morphology, texture, and levels of protein markers in any cell type. *Top*: Example images of M2 and M1 macrophages from either mouse (bone marrow derived macrophages, red: nuclei, blue: wheat germ agglutinin, green: actin) or human (macrophages derived from peripheral blood monocytes). Cellular morphology can be used to robustly discriminate macrophage phenotype in both mouse and human. Levels of protein markers (iNOS, STAT1) can also distinguish M1 and M2 phenotypes in human cells. *Bottom*: Sample computational workflow in high-content screening. In HCS, nuclei are often first segmented using either a DAPI or Hoechst stain, and then the surrounding cellular area is identified using a membrane and/or protein level stain. All quantified features can be examined at single cell resolution. Features can be analyzed individually or in an integrated, multidimensional fashion. For the latter, it is increasingly common to use methods from machine learning to robustly discriminate phenotypes. Methods include principal component analysis, k-means clustering, Gaussian mixture models, classification learners, etc. Assay robustness can be quantified using either SSMD or Z-factors.

Various phenotypes can be imaged in macrophages to search for agents that induce M1 polarization. Several previous studies have used levels of arginase 1 (Arg1) as a distinguishing marker of polarization, with Arg1 levels decreasing upon polarization from M2 to M1. However, recent work has shown that there is significant heterogeneity in Arg1 levels across M2 macrophages 7. Alternatively, the production of nitric oxide synthase 2 (iNOS, NOS2) expression, as wells as levels of various cytokines such as IL-12 or CXCL10, is more robust in M1 macrophages 24, 52. Staining for these markers upon polarization to M1 is likely a better approach for a direct M1/M2 comparison (**Figure 2**). These proteins are good targets to use for HCS, and validated antibodies are commercially available. Antibody staining is also possible in murine assays; though, it may be simpler to use primary bone marrow isolates from cytokine reporter mice where possible.

In addition to protein markers, other features pertaining to general cellular states may also help discriminate between macrophage states. Cell morphology is a particularly powerful tool to assess functionally relevant phenotypes 21. For example, M2 murine macrophages are typically elongated, with high length to width ratios, while M1 murine macrophages are round (**Figure 2**) 15, 53. In Rodell et. al., features pertaining to cellular shape were used to identify the TLR agonist resiquimod (R848) as an inducer of M1 polarization. Overall, an advantage with high-content microscopy is that general features of morphology can be simultaneously extracted while also staining for protein levels. Additionally, use of common dyes to label cellular components can allow for extraction of hundreds of parameters 54. This information can then be analyzed in parallel with protein level measurements in an integrated manner 55. Methods from machine learning will likely prove useful in analyzing high-content data, clustering macrophage subtypes, and scoring small molecules in screening assays (**Figure 2**) 56. Going forward, it will be interesting to evaluate whether other general cellular phenotypes (e.g., mitochondrial morphology) correlate with macrophage polarization. When using broader phenotype indicators, however, one must be careful as these cellular phenotypes are controlled by several regulators and pathways. In the case of morphological phenotypes for example, drugs that disrupt the cytoskeleton may score as false hits.

#### Co-culture and Trans-well Screening

Tumor-associated macrophages are just one component of the TME. In addition to other immune cell types (neutrophils, dendritic cells, granulocytes, lymphocytes), stromal and tumor cells can also influence macrophage phenotypes. As such, drugs perturbing these other components of the TME can indirectly affect macrophage activity. For example, cancer cells can release various factors (i.e., danger associated molecular patterns, DAMPs) into the local environment during immunogenic cell death (ICD) 57. These DAMPs directly activate myeloid cells through innate signaling pathways 14.

Given the complex interplay between cancer, stromal and immune cells, there is occasionally a need to develop co-culture screening assays in which immune cells are cultured with other cell types. Cells can be plated together, if contact interactions are necessary. In this case, use of fluorescent reporters and/or morphology can be used to distinguish cell types with a high-content imaging assay. Alternatively, if contact interactions are not necessary, one may use trans-well plates, in which one cell type is plated into the well, while the other(s) are on a semi-permeable insert, allowing for media exchange between the cell types.

Co-culture assays are particularly useful in rapidly profiling how cytotoxic chemotherapeutics affect macrophage polarization. It has long been known that cytotoxic chemotherapeutics and radiation therapy can induce inflammatory response in patients. One reported mechanism is that cytotoxic drugs induce ICD, causing the release of the protein HMGB1 and subsequent TLR4 pathway activation 58. Another emerging mechanism linked to several therapeutics is micronuclei formation. Micronuclei are fragmented pieces of DNA that arise in the cytosol 59, 60. Cytotoxic drugs, such as DNA-damaging agents, can cause micronuclei formation. These micronuclei then trigger the cGAS-STING pathway, expressed in various cancer types, resulting in pro-inflammatory cytokine secretion and macrophage polarization.

Macrophages also influence the therapeutic response of other immune cells to drugs. Microscopy based assays have been developed to quantitate the activity of CD8+ T cells, including how TAMs repress their cytotoxicity 61, 62, as well as how macrophages contribute to checkpoint blockade resistance through disadvantageous antibody uptake 37. It would be straightforward to develop high-content screening assays to identify therapeutics that perturb these processes. Given that it is now possible to image most types of immunocytes 63, numerous other co-culture screening assays can be developed to further identify macrophage immunomodulators.

#### Advanced Models

A number of more complex culture systems have been developed to more accurately mimic aspects of the *in vitro* environment. These include, for example, multicellular 3D culture platforms (e.g., spheroids, printed scaffolds) and microfluidic organs on a chip representing tumor-lymph node immune trafficking 64, 65. Some of these model systems, such as spheroids, are also compatible with all of the high-throughput assays mentioned above. These systems capture some aspects of the native environment, such as 3D architecture or select cell-cell interactions; however, they still often cannot fully recapitulate complex *in vivo* systems.

## *In Vivo* Imaging

One limitation of screening assays described above is the lack of a fully functional immune system. Intravital imaging approaches have thus emerged as a powerful method to study therapeutics in the presence of a fully intact immune environment, physiological forces, and a panoply of cells — all of which cannot be fully captured by model culture setups. Over the past decades, there has developed a veritable toolbox of methods for assessing the inflammatory state *in vivo*. These include methods to quantitate immune cell populations and phenotype through *in vivo* imaging. Successful execution of experiments requires good pairing of available animal models, imaging methods, and imaging probes to examine drug pharmacokinetics (PK) and pharmacodynamic (PD) outcomes. This section will review these important components of the intravital imaging toolbox, highlighting pertinent examples which examine myeloid cell activation or complex *in vivo* immune response in the tumor immune microenvironment and beyond.

### Animal Models

**Figure 3:**
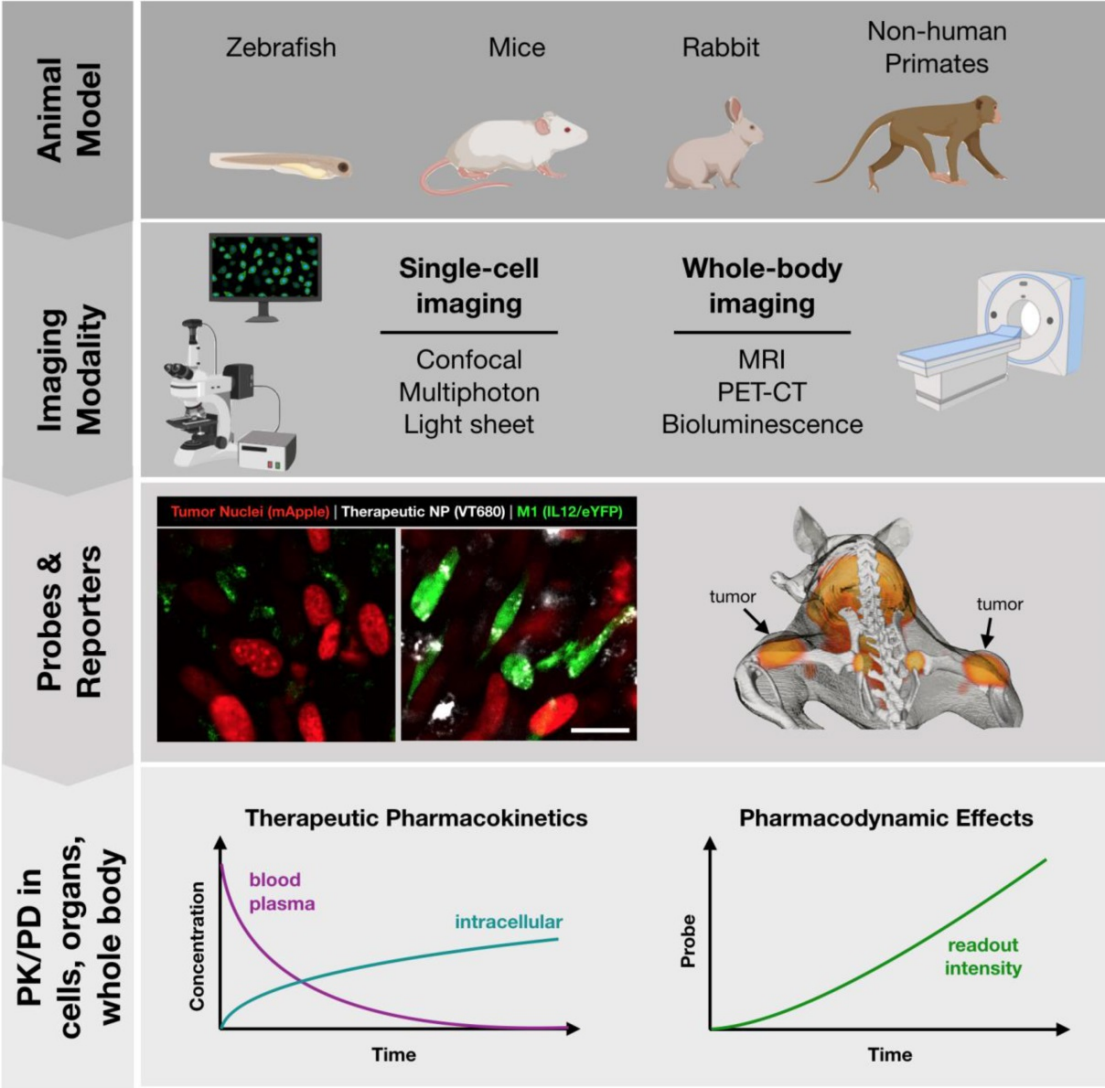
** Toolbox for development of successful *in vivo* imaging studies.** Development of *in vivo* imaging studies requires the selection and appropriate pairing of animal models, modes of imaging, and best readouts from appropriate imaging probes. Small animals (zebrafish, mice) are best suited for single-cell imaging approaches, such as cytokine reporters (imaged at left, Rodell unpublished data). Larger animal models (mice, rats, rabbits, primates) are best suited for whole-body imaging. Macrophage-targeted probes can indicate sites of active inflammation and dynamic response to therapy can be tracked longitudinally, such as by PET-CT probes (imaged at right; adapted with permission from 93, copyright 2018 American Chemical Society). These models allow for direct observation of therapeutic biodistribution to specific cell types (i.e., pharmacokinetics) as well as indicators of phenotypic changes such as cytokine production indicated by a eYFP reporter (i.e., pharmacodynamics).

A number of animal models are well suited to imaging at different scales, summarized in **Figure 3**. Zebrafish embryos are unique in that they are mostly transparent and can be readily imaged. Zebrafish have therefore been employed as a model to predict macrophage-associated biodistribution of nanoparticle therapeutics in the tumor environment 66, identify pH-dependent probes as indicators of macrophage activation 67 and to study macrophage polarization with fluorescent reporter systems 68 (**Table 3**). While there is broad conservation of macrophage functions and immune signaling across species, drug discovery applications may be limited by known differences in the regulatory pathways between zebrafish and higher level vertebrates 69-71, as well as limited tumor models.

Mice are the most commonly studied species. As a model system, they are relatively low-cost, easy to breed, and make an excellent platform for study due to the depth of prior characterization and availability of advanced tools such as transgenic and humanized mice. Indeed, a wide variety of genetically engineered variants are available, including knockout and reporter systems useful in drug screening. To aide in the identification of existing murine models, a number of resources are available such as the International Mouse Strain Resource (IMSR, http://www.findmice.org/) and Jackson Lab's Mouse Genome Informatics (MGI, http://www.informatics.jax.org/) database, amongst others. In addition, emergent tools such as CRISPR/Cas9 enable the rapid development of new mouse strains for study 72. Mice, whether wild-type or genetically engineered, are well suited to an array of imaging techniques, including both whole-body imaging and intravital microscopy to examine behavior at the single cell level. As discussed in subsequent sections, these capabilities are essential for PK/PD studies of therapeutic candidates.

As a complement to murine models, a number of larger animal models have been developed which are relevant for study of macrophage behavior. These include rabbit, pig, and primate models of atherosclerosis which are an excellent model for human disease 73-76, complementing the Apoe^-/-^ murine model 77. In the context of immuno-oncology, relevant large animal models include ovine lung cancer models for the study of alveolar macrophages and TAMs 78. Additionally, the oncopig is uniquely suited for the study of cancer immunotherapeutics in the presence of comorbid immune-related diseases, such as obesity and non-alcoholic steatohepatitis 79. Finally, non-human primates are a valuable model for general inflammatory response 80 and for the examination of macrophage-targeted imaging probes 74. For image-based examination, large animal models are best suited for whole-body imaging approaches.

### Imaging Modalities

There are a number of methods for imaging macrophage distribution and functional phenotype. Broadly, these can be classified into methods for single-cell and whole-body imaging (**Figure 3**). Methods of whole-body imaging include magnetic resonance imaging (MRI), nuclear imaging (PET-CT), and optical imaging (i.e., fluorescence mediated tomography, bioluminescence). Benefits of these methods include the potential for longitudinal tracking of response (over the course of hours to days), moderate throughput, and imaging deep within the body. These properties are particularly useful, as macrophage abundance is itself an important biomarker in diseases such as cancer, cardiovascular disease, and soft tissue injury. Over the years, a number of nanoparticle-based methods have been developed to track macrophages based on magnetic resonance imaging 81, 82; though, PET probes provide improved imaging sensitivity, such as for identification of metastatic sites.

In some instances, whole-body imaging can also provide information on the amplitude of immune activation. Many of these systems rely on targeting of receptors associated with polarizations, such as the folate (M1) and mannose receptor (M2) 83-85. More recently, Gambhir and colleagues have introduced a reporter system for optical imaging of macrophage polarization *in vivo*, where a luciferase reporter was placed under control of the Arg1 promoter, indicative of M2-like polarization. Adoptive transfer of these engineered macrophages into mice resulted in their migration to tumor sites as well as sites of soft tissue injury, identifiable by bioluminescence imaging 86. To enable moderate throughout screening for polarization *in vivo*, there are a number of optical probes commercially available. These include protease-activated near-IR fluorescence sensors (e.g., PerkinElmer's ProSense 680, a cathepsin activated fluorescent probe) that can be used to identify enzymatic activity associated with local inflammation 80, 87, 88.

While whole-body imaging is adept toward cell tracking and gross magnitude of immune response, single-cell imaging is uniquely able to observe cell interactions with each other and their environment that control bulk outcomes. For example, recent work by Uderhardt, et al. has shown that tissue resident macrophages rapidly envelop microlesions, blocking neutrophil activation and swarming. This short-term activation of macrophage cloaking behavior is crucial toward preventing neutrophil-associated inflammation, maintaining tissue homeostasis 89. Macrophages may also be activated in response to other insults, such as in the case of biomaterial implants. Recent examination of the foreign body response by nonlinear intravital microscopy has revealed M1 macrophage accumulation on the material surface that leads to development of multinuclear giant cells, implicated in release of VEGF, vascularization, and continued progression of fibrous encapsulation 90.

These examples highlight the ability of intravital microscopy to reveal single cell behaviors that drive tissue-level response. Similarly, there is a growing understanding that response of individual cells to pharmacological modulation contributes to overall response in cancer treatment 91. Examination of drug distribution (PK) and drug activity (PD) on individual cell populations in the TME therefore requires the use of intravital microscopy, and the application of these methods toward immunology has been recently reviewed 63, 91. Single-cell imaging *in vivo* relies upon optical imaging methods that include confocal fluorescence, multiphoton, and harmonic generation microscopy. While these methods uniquely allow for the direct visualization of drug location and cell phenotypic markers with excellent spatiotemporal resolution, limiting aspects include the timeframe of investigation possible (on the order of hours), high cost, and a high degree of technical ability needed for the experimental setup.

### Imaging Probes and Reporter Systems

Among the simplest methods of imaging macrophage location is their uptake of nanoparticles as imaging agents 81. A number of MRI and PET tracers have been described, and some of these are in clinical use or progressing towards clinical trials 92, 93. In contrast, assessment of macrophage phenotypes *in vivo* necessitates the use of imaging probes or reporter systems as a readout of therapeutic response. These include aforementioned probes for discrimination of phenotype based on relative expression of surface receptors as well as transporter proteins (TSPO, Slc18b1/SLC1881) 94, 95. A number of fluorescent probes have also been designed as indirect probes of macrophage activity, including pH indicating probes for ROS activity (e.g., PhagoGreen) 67. Another important indirect measure of macrophage activation is activity of secreted enzymes, including matrix metalloproteinases (MMPs) and cathepsins. Due to their inherent lytic activity, these enzymes lend themselves to the development of FRET probes. These classes of smart fluorescent probes have been thoroughly reviewed elsewhere 96.

The final method for assessing macrophage lineage and phenotype is the use of endogenous fluorescent reporter systems. Indeed, genetically engineered reporter mice are an invaluable tool in immunology and are an exceedingly good method for intravital imaging, compatible with an array of established tumor models. The reporters are engineered to express fluorescent reporter proteins alongside either cell lineage markers (e.g., CSF1R, MerTK) or cytokines well associated with polarization states (e.g., IL-12, Arg1). Myeloid phenotype reporters may be used to identify macrophages *in vivo* so that biodistribution of labeled drugs may be studied at the single cell level 91. On the other hand, polarization reporter systems can be used to directly observe changes in cell state following treatment. While reporter systems and methods of imaging macrophages by nanoparticle labeling have been more extensively reviewed elsewhere 63, 81, 97, a summary of pertinent models is provided in **Table 3**. A notable concern with reporter systems is somewhat limited specificity. For example, IL-10 is commonly associated with M2-like polarization. However, the cytokine is also highly expressed in T cell subsets, indicative of Th2-type response. Caution should therefore be taken to utilize a secondary phenotype indicator (e.g., fluorescently labeled ferumoxytol or dextran) if cytokine production is to be linked exclusively to macrophages.

### Pharmacokinetic (PK) and Pharmacodynamic (PD) Studies

These many imaging tools come together in the execution of PK/PD studies of promising therapeutic candidates, from which imaging datasets can yield quantifiable parameters to extract relevant PK and PD data including i) systemic half-life, ii) biodistribution to different organs, iii) cellular and subcellular distribution within the target tissue, and iv) magnitude of phenotype change. In order to examine all of these properties, a combination of independent imaging studies is often required. For initial studies of therapeutic biodistribution and efficacy, mouse models are the most appropriate as they lend themselves to both whole-body and single cell imaging approaches as well as follow-up studies on therapeutic efficacy.

In a typical experiment, drug candidates, such as those identified in cell based screens, can be examined either directly or following nanoformulation. Nanoformulation is a highly effective means of targeting therapeutics to myeloid cells, due in large part to their propensity to uptake a wide variety of materials on the nanoscale. Considerations for nanoparticle size, charge, and other properties to exploit this feature have been recently reviewed 98. For imaging purposes, labeling of the therapeutic drug or nanoparticle is typically required. For biodistribution at the whole-body level, development of radiotracers is common, such as through drug synthesis incorporating suitable isotopes for PET imaging 99, or by chelation of these isotopes with the nanoparticle 74, 93, 100, 101. The radiometric quantification of the therapeutic can then be monitored following administration by serial blood draw (to determine half-life) and by harvesting of tissues of interest (to determine organ biodistribution). Similar analysis can be accomplished by the development of fluorescently labeled drug derivatives as a companion imaging agent 102, by fluorescently labeling the nanoparticle 81, or by use of companion particles for imaging 103. For fluorescent tracers, similar quantification of therapeutic biodistribution proceeds by time-lapse microscopy of the vasculature (to determine half-life) and by image analysis of resected tissues (to determine organ biodistribution). Chief concerns for imaging agents are attenuation of fluorescence with depth as well as the potential for fluorescent drug conjugates to have altered subcellular distribution compared to the base compound. These issues may be addressed by use of long wavelength emitting fluorophores and screening of fluorophore conjugated libraries *in vitro*, respectively.

**Figure 4:**
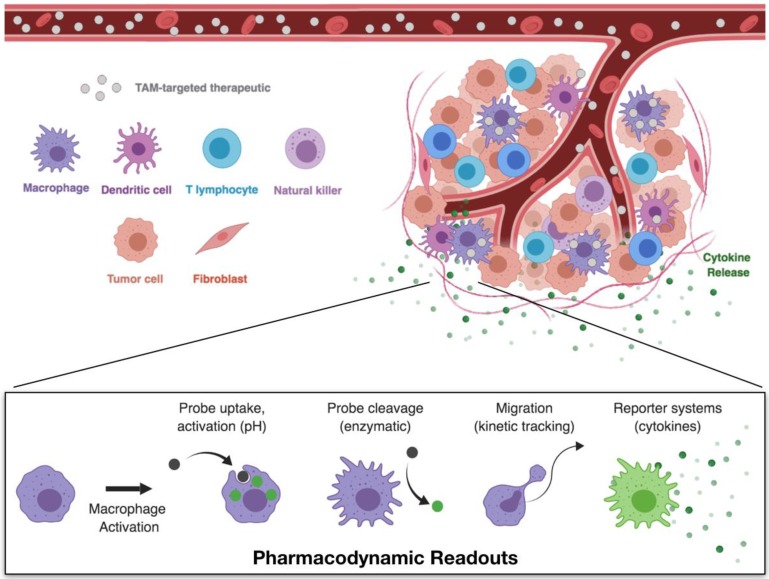
** Pharmacokinetic and pharmacodynamic studies through single-cell imaging.** Intravital microscopy allows for the simultaneous imaging of therapeutic biodistribution and macrophage phenotype. Following the administration of fluorescently labeled TAM therapeutics, compartment fluorescence may be monitored in the vasculature, interstitial space, or in specific cell types. These models therefore allow for direct observation of pharmacokinetics — the therapeutic biodistribution to specific tissues and cell types. Pairing of these techniques with appropriate model readouts for cell phenotype enable simultaneous monitoring of the pharmacodynamic effects — the direct observation of cell functions which are correlated with cell phenotype.

The TME is a complex environment, composed of a variety of host cell types. To examine drug distribution within the tumor and resulting drug effects on macrophage activation (**Figure 4**), intravital microscopy techniques are most appropriate. Techniques and equipment for intravital imaging of immune cell behavior have recently been expertly reviewed 63. For PK/PD studies, imaging may be performed on exteriorized organs, or by placement of optical imaging windows for visualization within skin, lung, brain, or other tissues 104, 105. Typically, tumors are implanted in these tissues by injection of suspended cells and allowed to grow until established and vascularized tumors are formed prior to treatment.

Experiments should be thoroughly planned before their execution to identify the necessary components to be visualized. Most confocal setups typically have up to four color channels, allowing visualization of labeled therapeutics (drugs, nanoparticles), structural components (extracellular matrix, vasculature), cell populations (tumor, stromal, or immune cells), and/or a probe or reporter system for the pharmacodynamic readout. Through pairing of appropriate labels, it is possible to discriminate between tissue and cell compartments which can be processed by quantitative image analysis to yield the desired PK/PD readouts. By establishing a delivery route (i.e., intravenous catheter) or injecting immediately preceding imaging, early distribution of the therapeutic through the vasculature and eventual vascular clearance can be assessed. Moreover, uptake by phagocytic cells in the TME can be quantified, as can the dynamic polarization response of these cells. Notably, one should understand that there may be a temporal lag in polarization responses (e.g., fluorescent reporter protein synthesis), as neither phenotypic changes or reporter protein synthesis are immediate processes, and may therefore require several hours to reach quantifiable levels.

Recent examples have highlighted the ability of these methods to unveil a novel understanding of drug PK/PD, where single cell behaviors are critical to overall therapeutic outcomes. These include the development of fluorescent prodrugs capable of selectively targeting and depleting M1 macrophages for rescue of a pro-regenerative response following injury 106. Macrophage depletion has similarly been examined in the TME, observing effects of anti-CSF1R blocking antibody therapy. In this instance, imaging revealed depletion of both TAM and M-DC phenotypes; however, therapeutic efficacy was modest 107. A contrasting approach to TAM depletion is TAM activation. We have recently demonstrated the development of a supramolecular nanocarrier 15, 108, which is rapidly uptaken by TAMs and acts as a vehicle for small molecule drug delivery through guest-host association 109. When paired with a potent TLR7/8 agonist (R848) for stimulation of TAMs, M1-like activation was indicated in IL-12 reporter models, resulting in therapeutic efficacy.

Additional studies have shown that macrophages are also an important mechanism of therapeutic action and resistance. For example, macrophage-associated drug uptake has been observed in the case of bisphosphonate, revealing TAM uptake as a driver of therapeutic efficacy 110. TAMs may also act as a therapeutic reservoir for continued, local release of anticancer therapeutics 111. Conversely, pharmacokinetic studies of anti-PD-1 have revealed an important resistance mechanism, whereby TAMs remove anti-PD-1 from the target T cells through Fcγ receptors and thereby undermine treatment 37. Efficacy of checkpoint therapies may therefore be improved through engineering of antibodies that avoid these resistance mechanisms. In sum, these studies highlight the ability of PK/PD studies to provide a clear picture of drug actions, and specifically how macrophages may influence therapeutic outcomes.

## Future Needs

New macrophage therapeutics will invariably emerge over the next few years given the central role of these cells in many human diseases and the strong rationale for developing them. From a translational perspective, there are two key questions: i) what clinical readouts are available to quantitate the efficacy of these new drugs and ii) what are some of the opportunities for other class specific cellular modulators?

Pharmacokinetic and pharmacodynamic readouts are essential in clinical trials 112. With respect to macrophage therapeutics, there exist at least three possible biomarkers: i) macrophage imaging using MRI (ferumoxytol) or PET (Macrin); ii) biopsies and fine needle aspirates (FNA); and iii) analysis of secreted proteins or extracellular vesicles in the peripheral blood. The most clinically advanced of these methods is macrophage MR imaging with super paramagnetic nanoparticles 81, 103, 113, which can be performed repeatedly in the same patient. More recently, PET based imaging agents with high affinity for macrophages have been developed 74, 93, 114. It is expected that some of these agents will enter clinical trials in 2020. Tumor biopsy by image guidance is currently a routinely performed method to sample cells. More recently, FNA methods have been developed that use smaller needles, have reduced morbidity, are better tolerated and allow immune cell profiling such as by cyclic imaging 48. Finally, it is well known that host immune cells shed extracellular vesicles into circulation; research is underway to identify these vesicles in peripheral blood 115. These methods could indeed provide a much needed window into the TME composition, as peripherally circulating immune cells have been show to poorly reflect the TME.

While we have mostly focused on macrophages in this topical review, a number of other myeloid derived immune cells are receiving increasing attention as therapeutic targets. Among these are myeloid derived suppressor cells (MDSC) 116, tumor-associated neutrophils (TAN) 1, dendritic cells (DC) 117 and granulocytes. It is hoped that some of the above described technology could be further adapted to testing emerging therapeutics for these immune cell populations as well. These approaches are important, both as monotherapies and in combination with standard of care checkpoint blockade therapies where improvement in clinical response rates would be hugely impactful.

## Figures and Tables

**Table 1:** ** Model cell lines for cell-based screens.** Physiological relevance of the cell source, difficulty of cell handling, and utility in high-throughput screening (HTS) assays are qualitatively scored (negligible: (-), low (+) to high (+++)).

Name	Description	Origin	Source	Comment		
				Relevance	Difficulty	HTS
RAW264.7 or J774.A.1	Immortalized murine macrophage cell line, with or without reporter	Mouse	ATCC, InvivoGen	++	-	+++
THP-1 or U937	Immortalized monocyte cell line, with or without reporter	Human	ATCC, InvivoGen	+	-	+++
HEK293 Reporter Cell lines	Immortalized human embryonic kidney cells, overexpressing pattern recognition receptor	Human host, Overexpressing mouse or human pathway	InvivoGen	-	-	+++ Immune pathway analysis only
BJ or BJ5ta	Fibroblasts isolated from foreskin (BJ); immortalized with telomerase (BJ5ta)	Human	ATCC	-	-	+++ Immune pathway analysis only
BMDM	Primary isolate	Mouse (B6)	Bone marrow B6 mice	+++	+	++
PBMC	From blood	Human	Blood Banks	+++	++	++
TAM	Primary MF isolate from TME	Murine tumors		+++	+++	+

**Table 2:** Assays for macrophage activating drug activity.

Assay Types	Description	Advantages	Disadvantages
Immune Cell Reporter Lines	THP-1 or RAW264.7 reporter assays	Probe multiple pathways Fast, cheap, easy	Physiological relevance Bulk measurements
HEK293 Reporter Cell lines	Test ligand binding to PRR of interest	Pathway specific Fast, cheap easy	Not examining macrophages directly
ELISAs and/or qPCRs for immune cytokines	Treat immune cells with drugs and measure cytokine levels	Probe multiple pathways Applicable to any cell type	Costly Bulk measurement Antibody dependent (ELISA)
High-content screening	Treat immune cells with drugs and stain for markers of interest	Physiological relevance Applicable to any cell type Single cell analysis	Costly More difficult Antibody dependent
Single cell RNAseq	Analysis of drug effects on genome, at single cell level	Comprehensive analysis of drug effect on many cell types Can identify heterogeneous effects	Very costly Not suitable for high-throughput screening Relatively new technology

**Table 3:** Common reporter systems for cell lineage and polarization.

Species	Reporter	Description & Notes	References
Zebra Fish	c-fms:mCherry	c-fms is also known as csf-1 receptor (CSF1R). Useful as a lineage reporter for macrophages.	118
	mpeg1:eGFP, mCherry	mpeg1 is macrophage expressed gene 1, a lineage reporter selective for macrophages vs neutrophils.	119
	mpeg1:mCherry, tnfa:eGFP	Double transgenic reporter for mpeg1 and tnfa (tumor necrosis factor alpha,), useful for identification of M1 activated macrophages.	68
Mouse	***Macrophage Lineage Reporter: used to identify cell type, such as in PK studies.***		
	CSF1R	Available as MacGreen, MacBlue.	120, 121
	CX3CR1^+/GFP^	Chemokine receptor also expressed by other immune cell types, including DC, NK, and T cells.	122
	CCR2^+/RFP^	Monocyte/macrophage associated chemokine receptor useful in examination of monocyte recruitment. Also expressed in NK, T cells.	123
	MerTK^+/GFP^	Receptor tyrosine kinase with excellent macrophage selectivity.	124
	Dye-conjugated dextran, particles, & antibodies	Administered before imaging to allow for macrophage uptake.	63, 81
	***Polarization Reporter: used to identify cell phenotype, such as in PD studies.***		
	IL-12-eYFP	M1-associated. p40-IRES-eYFP reporter, also indicates activated DCs distinguishable by higher expression levels.	125
	IFNγ-eYFP	M1-associated. Also referred to as GREAT mice. Also expressed in NK, T cells.	126
	Arg1-eYFP	M2-associated. Also referred to as YARG mice.	127
	IL-10-GFP	M2-associated. Numerous analogous reporters developed. Also expressed in DCs, Treg.	128

## References

[1] Engblom C, Pfirschke C, Zilionis R, Da Silva Martins J, Bos SA, Courties G (2017). Osteoblasts remotely supply lung tumors with cancer-promoting SiglecF^high^ neutrophils.

[2] Zilionis R, Engblom C, Pfirschke C, Savova V, Zemmour D, Saatcioglu HD (2019). Single-cell transcriptomics of human and mouse lung cancers reveals conserved myeloid populations across individuals and species. Immunity.

[3] Biswas SK, Mantovani A (2010). Macrophage plasticity and interaction with lymphocyte subsets: cancer as a paradigm. Nat Immunol.

[4] Ginhoux F, Schultze JL, Murray PJ, Ochando J, Biswas SK (2016). New insights into the multidimensional concept of macrophage ontogeny, activation and function. Nat Immunol.

[5] Noy R, Pollard JW (2014). Tumor-associated macrophages: from mechanisms to therapy. Immunity.

[6] Ruffell B, Affara NI, Coussens LM (2012). Differential macrophage programming in the tumor microenvironment. Trends Immunol.

[7] Arlauckas SP, Garren SB, Garris CS, Kohler RH, Oh J, Pittet MJ (2018). Arg1 expression defines immunosuppressive subsets of tumor-associated macrophages. Theranostics.

[8] Cassetta L, Kitamura T (2018). Targeting tumor-associated macrophages as a potential strategy to enhance the response to immune checkpoint inhibitors. Front Cell Dev Biol.

[9] van Dalen FJ, van Stevendaal MHME, Fennemann FL, Verdoes M, Ilina O (2018). Molecular repolarisation of tumour-associated macrophages. Molecules.

[10] Zeng Q, Jewell CM (2019). Directing toll-like receptor signaling in macrophages to enhance tumor immunotherapy. Curr Opin Biotechnol.

[11] Downey CM, Aghaei M, Schwendener RA, Jirik FR (2014). DMXAA causes tumor site-specific vascular disruption in murine non-small cell lung cancer, and like the endogenous non-canonical cyclic dinucleotide STING agonist, 2'3'-cGAMP, induces M2 macrophage repolarization. PLoS One.

[12] Kather JN, Halama N (2019). Harnessing the innate immune system and local immunological microenvironment to treat colorectal cancer.

[13] Kawai T, Akira S (2011). Toll-like receptors and their crosstalk with other innate receptors in infection and immunity. Immunity.

[14] King KR, Aguirre AD, Ye YX, Sun Y, Roh JD, Ng RP (2017). IRF3 and type I interferons fuel a fatal response to myocardial infarction. Nat Med.

[15] Rodell CB, Arlauckas SP, Cuccarese MF, Garris CS, Li R, Ahmed MS (2018). TLR7/8-agonist-loaded nanoparticles promote the polarization of tumour-associated macrophages to enhance cancer immunotherapy. Nat Biomed Eng.

[16] DeNardo DG, Brennan DJ, Rexhepaj E, Ruffell B, Shiao SL, Madden SF (2011). Leukocyte complexity predicts breast cancer survival and functionally regulates response to chemotherapy. Cancer Discov.

[17] DeNardo DG, Ruffell B (2019). Macrophages as regulators of tumour immunity and immunotherapy.

[18] Bhattacharya B, Chatterjee S, Devine WG, Kobzik L, Beeler AB, Porco JA (2016). Fine-tuning of macrophage activation using synthetic rocaglate derivatives. Sci Rep.

[19] Pryke KM, Abraham J, Sali TM, Gall BJ, Archer I, Liu A (2017). A Novel agonist of the TRIF pathway induces a cellular state refractory to replication of zika, chikungunya, and dengue viruses. MBio.

[20] Sali TM, Pryke KM, Abraham J, Liu A, Archer I, Broeckel R (2015). Characterization of a novel human-specific STING agonist that elicits antiviral activity against emerging alphaviruses. PLoS Pathog.

[21] Marklein RA, Lam J, Guvendiren M, Sung KE, Bauer SR (2018). Functionally-relevant morphological profiling: a tool to assess cellular heterogeneity. Trends Biotechnol.

[22] Jones DS, Jenney AP, Swantek JL, Burke JM, Lauffenburger DA, Sorger PK (2017). Profiling drugs for rheumatoid arthritis that inhibit synovial fibroblast activation. Nat Chem Biol.

[23] Koch PD, Miller HR, Yu G, Tallarico JA, Sorger PK, Wang Y (2018). A high content screen in macrophages identifies small molecule modulators of STING-IRF3 and NFkB signaling. ACS Chem Biol.

[24] Martinez FO, Gordon S, Locati M, Mantovani A (2006). Transcriptional profiling of the human monocyte-to-macrophage differentiation and polarization: new molecules and patterns of gene expression. J Immunol.

[25] Scotton CJ, Martinez FO, Smelt MJ, Sironi M, Locati M, Mantovani A (2005). Transcriptional profiling reveals complex regulation of the monocyte IL-1 beta system by IL-13. J Immunol.

[26] Spiller KL, Wrona EA, Romero-Torres S, Pallotta I, Graney PL, Witherel CE (2016). Differential gene expression in human, murine, and cell line-derived macrophages upon polarization. Exp Cell Res.

[27] van Wilgenburg B, Browne C, Vowles J, Cowley SA (2013). Efficient, long term production of monocyte-derived macrophages from human pluripotent stem cells under partly-defined and fully-defined conditions. PLoS One.

[28] Haenseler W, Sansom SN, Buchrieser J, Newey SE, Moore CS, Nicholls FJ (2017). A highly efficient human pluripotent stem cell microglia model displays a neuronal-co-culture-specific expression profile and inflammatory response. Stem Cell Reports.

[29] Lopez-Yrigoyen M, Fidanza A, Cassetta L, Axton RA, Taylor AH, Meseguer-Ripolles J (2018). A human iPSC line capable of differentiating into functional macrophages expressing ZsGreen: a tool for the study and in vivo tracking of therapeutic cells. Philos Trans R Soc Lond B Biol Sci.

[30] Senju S, Koba C, Haruta M, Matsunaga Y, Matsumura K, Haga E (2014). Application of iPS cell-derived macrophages to cancer therapy. Oncoimmunology.

[31] Genin M, Clement F, Fattaccioli A, Raes M, Michiels C (2015). M1 and M2 macrophages derived from THP-1 cells differentially modulate the response of cancer cells to etoposide. BMC Cancer.

[32] Park EK, Jung HS, Yang HI, Yoo MC, Kim C, Kim KS (2007). Optimized THP-1 differentiation is required for the detection of responses to weak stimuli. Inflamm Res.

[33] Ni G, Chen S, Yuan J, Cavezza SF, Wei MQ, Li H (2019). Comparative proteomic study reveals the enhanced immune response with the blockade of interleukin 10 with anti-IL-10 and anti-IL-10 receptor antibodies in human U937 cells. PLoS One.

[34] Konno H, Konno K, Barber GN (2013). Cyclic dinucleotides trigger ULK1 (ATG1) phosphorylation of STING to prevent sustained innate immune signaling. Cell.

[35] Weischenfeldt J, Porse B (2008). Bone marrow-derived macrophages (BMM): isolation and applications. Cold Spring Harb Protoc.

[36] Ying W, Cheruku PS, Bazer FW, Safe SH, Zhou B (2013). Investigation of macrophage polarization using bone marrow derived macrophages.

[37] Arlauckas SP, Garris CS, Kohler RH, Kitaoka M, Cuccarese MF, Yang KS (2017). In vivo imaging reveals a tumor-associated macrophage-mediated resistance pathway in anti-PD-1 therapy. Sci Transl Med.

[38] De Nardo D, Kalvakolanu DV, Latz E (2018). Immortalization of murine bone marrow-derived macrophages. Methods Mol Biol.

[39] Taciak B, Białasek M, Braniewska A, Sas Z, Sawicka P, Kiraga Ł et al (2018). Evaluation of phenotypic and functional stability of RAW 264.7 cell line through serial passages. PLoS One.

[40] Hemmi H, Kaisho T, Takeuchi O, Sato S, Sanjo H, Hoshino K (2002). Small anti-viral compounds activate immune cells via the TLR7 MyD88-dependent signaling pathway. Nat Immunol.

[41] Cheng K, Gao M, Godfroy JI, Brown PN, Kastelowitz N, Yin H (2015). Specific activation of the TLR1-TLR2 heterodimer by small-molecule agonists. Sci Adv.

[42] Jerby-Arnon L, Shah P, Cuoco MS, Rodman C, Su MJ, Melms JC (2018). A cancer cell program promotes T cell exclusion and resistance to checkpoint blockade. Cell.

[43] Tirosh I, Izar B, Prakadan SM, Wadsworth MH, Treacy D, Trombetta JJ (2016). Dissecting the multicellular ecosystem of metastatic melanoma by single-cell RNA-seq. Science.

[44] Villani AC, Satija R, Reynolds G, Sarkizova S, Shekhar K, Fletcher J (2017). Single-cell RNA-seq reveals new types of human blood dendritic cells, monocytes, and progenitors. Science.

[45] Bodenmiller B, Zunder ER, Finck R, Chen TJ, Savig ES, Bruggner RV (2012). Multiplexed mass cytometry profiling of cellular states perturbed by small-molecule regulators. Nat Biotechnol.

[46] Ding M, Clark R, Bardelle C, Backmark A, Norris T, Williams W (2018). Application of high-throughput flow cytometry in early drug discovery: an AstraZeneca perspective. SLAS Discov.

[47] Tang Y, Xie T, Florian S, Moerke N, Shamu C, Benes C (2013). Differential determinants of cancer cell insensitivity to antimitotic drugs discriminated by a one-step cell imaging assay. J Biomol Screen.

[48] Giedt RJ, Pathania D, Carlson JCT, McFarland PJ, Del Castillo AF, Juric D (2018). Single-cell barcode analysis provides a rapid readout of cellular signaling pathways in clinical specimens. Nat Commun.

[49] Lin JR, Fallahi-Sichani M, Sorger PK (2015). Highly multiplexed imaging of single cells using a high-throughput cyclic immunofluorescence method. Nat Commun.

[50] Goltsev Y, Samusik N, Kennedy-Darling J, Bhate S, Hale M, Vazquez G (2018). Deep profiling of mouse splenic architecture with CODEX multiplexed imaging. Cell.

[51] Ullal AV, Peterson V, Agasti SS, Tuang S, Juric D, Castro CM (2014). Cancer cell profiling by barcoding allows multiplexed protein analysis in fine-needle aspirates. Sci Transl Med.

[52] Rostam HM, Reynolds PM, Alexander MR, Gadegaard N, Ghaemmaghami AM (2017). Image based machine learning for identification of macrophage subsets. Sci Rep.

[53] Nishio M, Urakawa N, Shigeoka M, Takase N, Ichihara Y, Arai N (2016). Software-assisted morphometric and phenotype analyses of human peripheral blood monocyte-derived macrophages induced by a microenvironment model of human esophageal squamous cell carcinoma. Pathol Int.

[54] Bray MA, Gustafsdottir SM, Rohban MH, Singh S, Ljosa V, Sokolnicki KL (2017). A dataset of images and morphological profiles of 30 000 small-molecule treatments using the Cell Painting assay. Gigascience.

[55] Perlman ZE, Slack MD, Feng Y, Mitchison TJ, Wu LF, Altschuler SJ (2004). Multidimensional drug profiling by automated microscopy. Science.

[56] Bray MA, Carpenter AE (2018). Quality control for high-throughput imaging experiments using machine learning in Cellprofiler. Methods Mol Biol.

[57] Galluzzi L, Buqué A, Kepp O, Zitvogel L, Kroemer G (2017). Immunogenic cell death in cancer and infectious disease. Nat Rev Immunol.

[58] Pfirschke C, Engblom C, Rickelt S, Cortez-Retamozo V, Garris C, Pucci F (2016). Immunogenic chemotherapy sensitizes tumors to checkpoint blockade therapy. Immunity.

[59] Dou Z, Ghosh K, Vizioli MG, Zhu J, Sen P, Wangensteen KJ (2017). Cytoplasmic chromatin triggers inflammation in senescence and cancer. Nature.

[60] Harding SM, Benci JL, Irianto J, Discher DE, Minn AJ, Greenberg RA (2017). Mitotic progression following DNA damage enables pattern recognition within micronuclei. Nature.

[61] Halle S, Halle O, Förster R (2017). Mechanisms and dynamics of T cell-mediated cytotoxicity in vivo. Trends Immunol.

[62] Kitamura T, Doughty-Shenton D, Pollard JW, Carragher NO (2019). Real time detection of in vitro tumor cell apoptosis induced by CD8+ T cells to study immune suppressive functions of tumor-infiltrating myeloid cells. J Vis Exp.

[63] Pittet MJ, Garris CS, Arlauckas SP, Weissleder R (2018). Recording the wild lives of immune cells. Sci Immunol.

[64] Shim S, Belanger MC, Harris AR, Munson JM, Pompano RR (2019). Two-way communication between ex vivo tissues on a microfluidic chip: application to tumor-lymph node interaction. Lab Chip.

[65] Katt ME, Placone AL, Wong AD, Xu ZS, Searson PC (2016). In vitro tumor models: advantages, disadvantages, variables, and selecting the right platform. Front Bioeng Biotechnol.

[66] Campbell F, Bos FL, Sieber S, Arias-Alpizar G, Koch BE, Huwyler J (2018). Directing nanoparticle biodistribution through evasion and exploitation of Stab2-dependent nanoparticle uptake. ACS Nano.

[67] Vázquez-Romero A, Kielland N, Arévalo MJ, Preciado S, Mellanby RJ, Feng Y (2013). Multicomponent reactions for de novo synthesis of BODIPY probes: in vivo imaging of phagocytic macrophages. J Am Chem Soc.

[68] Nguyen-Chi M, Laplace-Builhe B, Travnickova J, Luz-Crawford P, Tejedor G, Phan QT (2015). Identification of polarized macrophage subsets in zebrafish. Elife.

[69] Ge R, Zhou Y, Peng R, Wang R, Li M, Zhang Y (2015). Conservation of the STING-Mediated Cytosolic DNA Sensing Pathway in Zebrafish. J Virol.

[70] Li S, Lu LF, Li ZC, Zhang C, Zhou XY, Zhou Y (2019). Zebrafish MVP Recruits and Degrades TBK1 To Suppress IFN Production. J Immunol.

[71] Ma JX, Li JY, Fan DD, Feng W, Lin AF, Xiang LX (2018). Identification of DEAD-Box RNA Helicase DDX41 as a Trafficking Protein That Involves in Multiple Innate Immune Signaling Pathways in a Zebrafish Model. Front Immunol.

[72] Khaled WT, Liu P (2014). Cancer mouse models: past, present and future. Semin Cell Dev Biol.

[73] Fan J, Kitajima S, Watanabe T, Xu J, Zhang J, Liu E (2015). Rabbit models for the study of human atherosclerosis: from pathophysiological mechanisms to translational medicine. Pharmacology & therapeutics.

[74] Keliher EJ, Ye YX, Wojtkiewicz GR, Aguirre AD, Tricot B, Senders ML (2017). Polyglucose nanoparticles with renal elimination and macrophage avidity facilitate PET imaging in ischaemic heart disease. Nat Commun.

[75] Lameijer M, Binderup T, van Leent MMT, Senders ML, Fay F, Malkus J (2018). Efficacy and safety assessment of a TRAF6-targeted nanoimmunotherapy in atherosclerotic mice and non-human primates. Nat Biomed Eng.

[76] Pérez-Medina C, Binderup T, Lobatto ME, Tang J, Calcagno C, Giesen L (2016). In vivo PET imaging of HDL in multiple atherosclerosis models. ACC Cardiovasc Imaging.

[77] Sasso GL, Schlage WK, Boué S, Veljkovic E, Peitsch MC, Hoeng J (2016). The Apoe-/- mouse model: a suitable model to study cardiovascular and respiratory diseases in the context of cigarette smoke exposure and harm reduction. J Transl Med.

[78] Gray ME, Meehan J, Sullivan P, Marland JRK, Greenhalgh SN, Gregson R (2019). Ovine pulmonary adenocarcinoma: a unique model to improve lung cancer research. Front Oncol.

[79] Schachtschneider KM, Schwind RM, Newson J, Kinachtchouk N, Rizko M, Mendoza-Elias N (2017). The oncopig cancer model: an innovative large animal translational oncology platform. Front Oncol.

[80] Vegas AJ, Veiseh O, Doloff JC, Ma M, Tam HH, Bratlie K (2016). Combinatorial hydrogel library enables identification of materials that mitigate the foreign body response in primates. Nat Biotechnol.

[81] Weissleder R, Nahrendorf M, Pittet MJ (2014). Imaging macrophages with nanoparticles. Nat Mater.

[82] Ahrens ET, Bulte JW (2013). Tracking immune cells in vivo using magnetic resonance imaging. Nat Rev Immunol.

[83] Baker DW, Zhou J, Tsai Y-T, Patty KM, Weng H, Tang EN (2014). Development of optical probes for in vivo imaging of polarized macrophages during foreign body reactions. Acta Biomater.

[84] Jager NA, Westra J, Golestani R, Van Dam GM, Low PS, Tio RA (2014). Folate receptor-β imaging using 99mTc-folate to explore distribution of polarized macrophage populations in human atherosclerotic plaque. J Nucl Med.

[85] Zhou J, Tsai Y-T, Weng H, Baker DW, Tang L (2011). Real time monitoring of biomaterial-mediated inflammatory responses via macrophage-targeting NIR nanoprobes. Biomaterials.

[86] Aalipour A, Chuang H-Y, Murty S, D'Souza AL, Park S-m, Gulati GS (2019). Engineered immune cells as highly sensitive cancer diagnostics.

[87] Weissleder R, Tung CH, Mahmood U, Bogdanov A (1999). In vivo imaging of tumors with protease-activated near-infrared fluorescent probes. Nat Biotechnol.

[88] Bremer C, Tung CH, Weissleder R (2001). In vivo molecular target assessment of matrix metalloproteinase inhibition. Nat Med.

[89] Uderhardt S, Martins AJ, Tsang JS, Lämmermann T, Germain RN (2019). Resident macrophages cloak tissue microlesions to prevent neutrophil-driven inflammatory damage. Cell.

[90] Dondossola E, Holzapfel BM, Alexander S, Filippini S, Hutmacher DW, Friedl P (2017). Examination of the foreign body response to biomaterials by nonlinear intravital microscopy. Nat Biomed Eng.

[91] Miller MA, Weissleder R (2017). Imaging of anticancer drug action in single cells. Nat Rev Cancer.

[92] Fernández-Friera L, Fuster V, López-Melgar B, Oliva B, Sánchez-González J, Macías A (2019). Vascular inflammation in subclinical atherosclerosis detected by hybrid PET/MRI. J Am Coll Cardiol.

[93] Kim HY, Li R, Ng TSC, Courties G, Rodell CB, Prytyskach M (2018). Quantitative imaging of tumor associated macrophages and their response to therapy using 64Cu-labeled macrin. ACS Nano.

[94] Gaemperli O, Shalhoub J, Owen DRJ, Lamare F, Johansson S, Fouladi N (2011). Imaging intraplaque inflammation in carotid atherosclerosis with 11C-PK11195 positron emission tomography/computed tomography. Eur Heart J.

[95] Park S-J, Kim B, Choi S, Balasubramaniam S, Lee S-C, Lee JY (2019). Imaging inflammation using an activated macrophage probe with Slc18b1 as the activation-selective gating target. Nat Commun.

[96] Fernández A, Vendrell M (2016). Smart fluorescent probes for imaging macrophage activity. Chem Soc Rev.

[97] Croxford AL, Buch T (2011). Cytokine reporter mice in immunological research: perspectives and lessons learned. Immunology.

[98] Behzadi S, Serpooshan V, Tao W, Hamaly MA, Alkawareek MY, Dreaden EC (2017). Cellular uptake of nanoparticles: journey inside the cell. Chem Soc Rev.

[99] Weber WA (2006). Positron emission tomography as an imaging biomarker. J Clin Oncol.

[100] Keliher EJ, Yoo J, Nahrendorf M, Lewis JS, Marinelli B, Newton A (2011). 89Zr-labeled dextran nanoparticles allow in vivo macrophage imaging. Bioconjug Chem.

[101] Locke LW, Mayo MW, Yoo AD, Williams MB, Berr SS (2012). PET imaging of tumor associated macrophages using mannose coated 64Cu liposomes. Biomaterials.

[102] Thurber GM, Yang KS, Reiner T, Kohler RH, Sorger P, Mitchison T (2013). Single-cell and subcellular pharmacokinetic imaging allows insight into drug action in vivo. Nat Commun.

[103] Miller MA, Gadde S, Pfirschke C, Engblom C, Sprachman MM, Kohler RH (2015). Predicting therapeutic nanomedicine efficacy using a companion magnetic resonance imaging nanoparticle. Sci Transl Med.

[104] Alieva M, Ritsma L, Giedt RJ, Weissleder R, van Rheenen J (2014). Imaging windows for long-term intravital imaging: General overview and technical insights. Intravital.

[105] Entenberg D, Voiculescu S, Guo P, Borriello L, Wang Y, Karagiannis GS (2018). A permanent window for the murine lung enables high-resolution imaging of cancer metastasis. Nat Methods.

[106] Fernandez A, Vermeren M, Humphries D, Subiros-Funosas R, Barth N, Campana L (2017). Chemical modulation of in vivo macrophage function with subpopulation-specific fluorescent prodrug conjugates. ACS Cent Sci.

[107] Lohela M, Casbon AJ, Olow A, Bonham L, Branstetter D, Weng N (2014). Intravital imaging reveals distinct responses of depleting dynamic tumor-associated macrophage and dendritic cell subpopulations. Proc Natl Acad Sci U S A.

[108] Ahmed MS, Rodell CB, Hulsmans M, Kohler RH, Aguirre A, Nahrendorf M (2019). A supramolecular nanocarrier for delivery of amiodarone anti-arrhythmic therapy to the heart. Bioconjug Chem.

[109] Rodell CB, Mealy JE, Burdick JA (2015). Supramolecular guest-host interactions for the preparation of biomedical materials. Bioconjug Chem.

[110] Junankar S, Shay G, Jurczyluk J, Ali N, Down J, Pocock N (2015). Real-time intravital imaging establishes tumor-associated macrophages as the extraskeletal target of bisphosphonate action in cancer. Cancer Discov.

[111] Miller MA, Zheng YR, Gadde S, Pfirschke C, Zope H, Engblom C (2015). Tumour-associated macrophages act as a slow-release reservoir of nano-therapeutic Pt(IV) pro-drug. Nat Commun.

[112] Miller MA, Weissleder R (2017). Imaging the pharmacology of nanomaterials by intravital microscopy: Toward understanding their biological behavior. Adv Drug Deliv Rev.

[113] Gaglia JL, Harisinghani M, Aganj I, Wojtkiewicz GR, Hedgire S, Benoist C (2015). Noninvasive mapping of pancreatic inflammation in recent-onset type-1 diabetes patients. Proc Natl Acad Sci U S A.

[114] Majmudar MD, Yoo J, Keliher EJ, Truelove JJ, Iwamoto Y, Sena B (2013). Polymeric nanoparticle PET/MR imaging allows macrophage detection in atherosclerotic plaques. Circ Res.

[115] Shao H, Im H, Castro CM, Breakefield X, Weissleder R, Lee H (2018). New technologies for analysis of extracellular vesicles. Chem Rev.

[116] Fleming V, Hu X, Weber R, Nagibin V, Groth C, Altevogt P (2018). Targeting myeloid-derived suppressor cells to bypass tumor-induced immunosuppression. Front Immunol.

[117] Garris CS, Arlauckas SP, Kohler RH, Trefny MP, Garren S, Piot C (2018). Successful anti-PD-1 cancer immunotherapy requires T cell-dendritic cell crosstalk involving the cytokines IFN-γ and IL-12. Immunity.

[118] Gray C, Loynes CA, Whyte MK, Crossman DC, Renshaw SA, Chico TJ (2011). Simultaneous intravital imaging of macrophage and neutrophil behaviour during inflammation using a novel transgenic zebrafish. Thromb Haemost.

[119] Ellett F, Pase L, Hayman JW, Andrianopoulos A, Lieschke GJ (2011). mpeg1 promoter transgenes direct macrophage-lineage expression in zebrafish. Blood.

[120] Sauter KA, Pridans C, Sehgal A, Bain CC, Scott C, Moffat L (2014). The MacBlue binary transgene (csf1r-gal4VP16/UAS-ECFP) provides a novel marker for visualisation of subsets of monocytes, macrophages and dendritic cells and responsiveness to CSF1 administration. PLoS One.

[121] Wyckoff JB, Wang Y, Lin EY, Li JF, Goswami S, Stanley ER (2007). Direct visualization of macrophage-assisted tumor cell intravasation in mammary tumors. Cancer Res.

[122] Auffray C, Fogg D, Garfa M, Elain G, Join-Lambert O, Kayal S (2007). Monitoring of blood vessels and tissues by a population of monocytes with patrolling behavior. Science.

[123] Saederup N, Cardona AE, Croft K, Mizutani M, Cotleur AC, Tsou CL (2010). Selective chemokine receptor usage by central nervous system myeloid cells in CCR2-red fluorescent protein knock-in mice. PLoS One.

[124] Mohan JF, Kohler RH, Hill JA, Weissleder R, Mathis D, Benoist C (2017). Imaging the emergence and natural progression of spontaneous autoimmune diabetes. Proc Natl Acad Sci U S A.

[125] Reinhardt RL, Hong S, Kang SJ, Wang ZE, Locksley RM (2006). Visualization of IL-12/23p40 in vivo reveals immunostimulatory dendritic cell migrants that promote Th1 differentiation. J Immunol.

[126] Reinhardt RL, Liang HE, Locksley RM (2009). Cytokine-secreting follicular T cells shape the antibody repertoire. Nat Immunol.

[127] Reese TA, Liang HE, Tager AM, Luster AD, Van Rooijen N, Voehringer D (2007). Chitin induces accumulation in tissue of innate immune cells associated with allergy. Nature.

[128] Bouabe H (2012). Cytokine reporter mice: the special case of IL-10. Scand J Immunol.

